# Xylem Parenchyma—Role and Relevance in Wood Functioning in Trees

**DOI:** 10.3390/plants10061247

**Published:** 2021-06-19

**Authors:** Aleksandra Słupianek, Alicja Dolzblasz, Katarzyna Sokołowska

**Affiliations:** Department of Plant Developmental Biology, Faculty of Biological Sciences, University of Wrocław, Kanonia 6/8, 50-328 Wrocław, Poland; alicja.dolzblasz@uwr.edu.pl (A.D.); katarzyna.sokolowska@uwr.edu.pl (K.S.)

**Keywords:** carbohydrates storage, CODIT, contact cells, embolism, hydraulic conductance, trees, vessel-associated cells, water storage, xylem parenchyma

## Abstract

Woody plants are characterised by a highly complex vascular system, wherein the secondary xylem (wood) is responsible for the axial transport of water and various substances. Previous studies have focused on the dead conductive elements in this heterogeneous tissue. However, the living xylem parenchyma cells, which constitute a significant functional fraction of the wood tissue, have been strongly neglected in studies on tree biology. Although there has recently been increased research interest in xylem parenchyma cells, the mechanisms that operate in these cells are poorly understood. Therefore, the present review focuses on selected roles of xylem parenchyma and its relevance in wood functioning. In addition, to elucidate the importance of xylem parenchyma, we have compiled evidence supporting the hypothesis on the significance of parenchyma cells in tree functioning and identified the key unaddressed questions in the field.

## 1. Introduction

Vascular plants have developed a sophisticated and efficient transport system that supplies water and various other substances, including photoassimilates, ions, and hormones, to all plant organs. The vascular system comprises two main tissue types, phloem and xylem, which are classified into primary, originating from the procambium, and secondary, originating from the cambium [1,2,3]. The vascular cambium is found in plants with secondary growth and can continuously produce secondary phloem outward and secondary xylem (wood) inward once formed. Consequently, the lateral thickening of the stem allows plants to obtain exceptional height and radial growth, which, in turn, enable the plant to access better light conditions [3,4].

The structure and complexity of the vascular system vary, and are particularly advanced in woody plants [1,5]. The vascular system plays an important role in the regulation of various developmental and physiological processes, and owing to the long lifespan and large size of woody plants, it is crucial for the efficient long-distance communication between the distantly positioned roots and the tree’s canopy [5,6,7,8]. Thus, the present review specifically concentrates on the secondary xylem; readers interested in the secondary phloem can refer to other review papers (e.g., [9,10,11,12,13]).

### 1.1. Structure of the Secondary Xylem (Wood)

Mature xylem tissue comprises three main cell types: tracheary elements (conductive elements or conduits), fibres, and parenchyma cells. Tracheary elements, which are dead cells, are the major type of wood cells arranged and connected axially to form a long route for transporting xylem sap. These elements can also resist large negative pressure because of their thick, lignified secondary cell walls [1,2,14]. Based on the type of joining, two types of xylem conduits have been identified: tracheids and vessel elements [15]. 

Tracheids are relatively narrow conductive elements, connected to each other via holes in the secondary cell wall, called bordered pits. Tracheids are a major component of the wood in gymnosperms (conifers) and are occasionally found in the wood of angiosperms. In addition to transporting xylem sap, tracheids strengthen the wood structure in conifers, which lack the supporting cells, fibres [1,16,17,18]. The other type of conductive elements, which are connected via perforation plates (open end walls between adjacent cells), are called vessel elements. Vessel elements are the major conductive cell type in angiosperms, usually wider in diameter than tracheids and arranged axially, one above the other, to form long tubes called vessels [5,16]. In addition to the axial transport in tracheary elements, xylem sap is transported via interconduit pits, which allow for the lateral flow of solutes between adjacent conductive elements. Additionally, pits can connect conduits with the neighbouring non-tracheary elements, xylem parenchyma cells [19,20].

The supporting elements in angiosperm wood are fibres, which are long, mostly dead cells with lignified, thick secondary cell walls [1]. Notably, when fibres still possess living protoplasts, they also participate in carbohydrate storage and mobilisation [21,22]. The last type of wood cells are xylem parenchyma cells, which may be oriented axially or radially, with the latter forming rays. Although these cells typically have relatively thin secondary cell walls, usually lignified, they maintain their metabolic activity for many years [1,23,24] and perform diverse crucial functions for wood and tree functioning (e.g., [25,26,27,28,29]). 

Furthermore, two types of wood can be found in the tree stem: sapwood and heartwood. Sapwood is found in the outer wood zone, which is the physiologically active part of the secondary xylem. It contains living cells and it is the region where xylem sap is transported within tracheary elements. In contrast, heartwood localises in the inner wood zone, which is the inactive wood fraction devoid of living cells and where xylem sap transport does not occur. The transition from sapwood to heartwood occurs as the tree ages [30,31].

**Figure 1:**
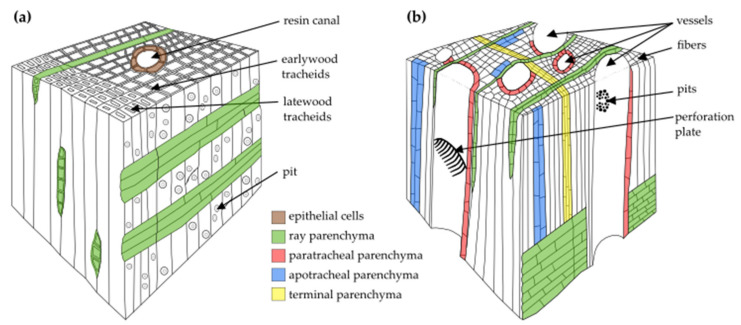
Schematic representation of softwood (**a**) and hardwood (**b**). Due to the species variety, the diagrams were simplified, and only selected cell types were marked. Moreover, cell proportions might not be accurate.

The secondary xylem in gymnosperms (also called softwood) is less complex than that in angiosperms (also called hardwood). Gymnosperm wood primarily consists of tracheids (reaching up to 90% in volume) and only a small amount of xylem parenchyma cells, often limited to rays [15,16,18], whereas angiosperm wood consists of vessel elements, tracheids, fibres, and abundant xylem parenchyma fractions [1]. Moreover, in some groups of conifers, resin canals can also be identified [18] (Figure 1).

### 1.2. Xylem Parenchyma

Xylem parenchyma cells are characterised by simple pits, where numerous plasmodesmata are localised. Thus, they are well connected and form a 3D network of interconnected protoplasts belonging to both the axial and radial systems of the secondary xylem [1,32,33,34,35,36,37]. Moreover, parenchyma cells constitute a connection bridge, which extends from the xylem to the phloem and forms a functional association between these tissues. It has been reported that the transpiration stream in xylem conduits is directly connected with the phloem tissue via ray parenchyma [38,39,40,41,42]. 

**Figure 2:**
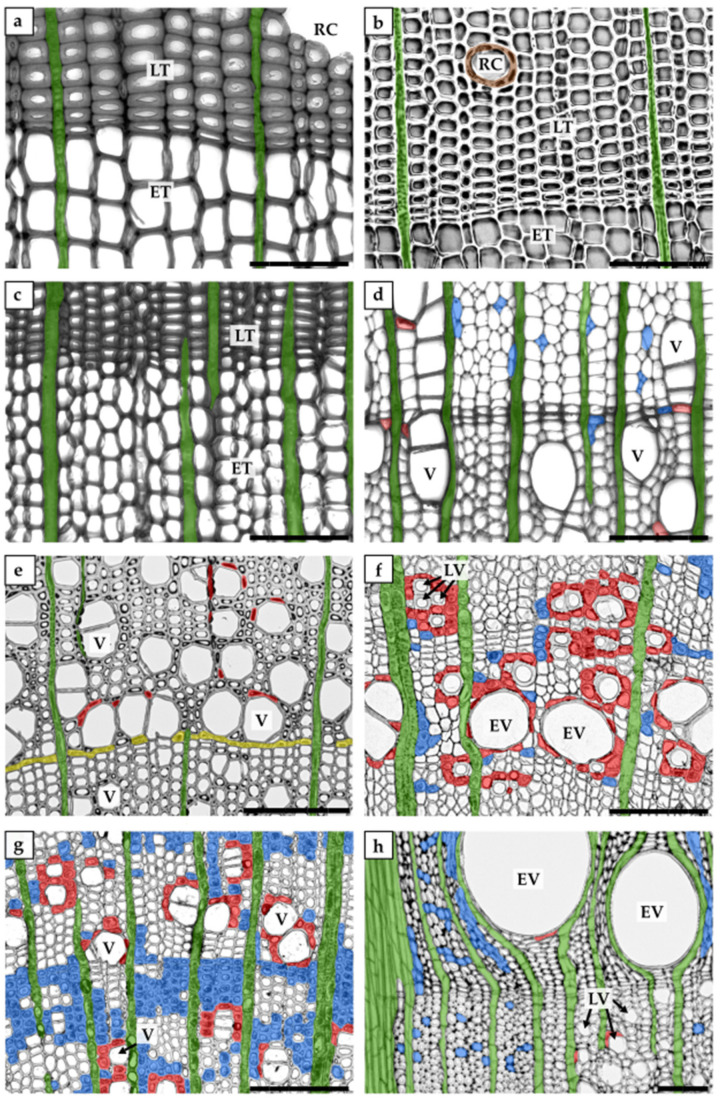
Types of xylem parenchyma cells of selected gymnosperm and angiosperm species. Transverse sections of the secondary xylem of gymnosperm trees (**a**—*Pinus* sp., **b**—*Picea* sp., **c**—*Abies* sp.) and angiosperm trees (**d**—*Aesculus* sp., **e**—*Populus* sp., **f**—*Fraxinus* sp., **g**—*Acer* sp., **h**—*Quercus* sp.). Different types of xylem parenchyma are marked with various colours: ray parenchyma with green, paratracheal parenchyma with red, apotracheal parenchyma with blue, and terminal parenchyma with yellow. Epithelial cells in *Picea* sp. wood are marked with brown. Mature wood is shown in (a,b,c,d,h), and juvenile wood in (e,f,g). Abbreviations: ET—earlywood tracheids, LT—latewood tracheids, RC—resin canal, EV—earlywood vessel, LV—latewood vessel, V—vessel. Scale bars—100 µm.

In the context of xylem parenchyma classification, various terminologies have been used; therefore, we have attempted to use terminology consistent with that used in the original papers discussed in this review. Based on the cell orientation in wood, the xylem parenchyma is classified into axial or ray parenchyma (Figure 1 and Figure 2, Table 1). In the axial parenchyma, two major fractions are distinguished: the paratracheal parenchyma, which abuts conductive elements, and the apotracheal parenchyma, which is usually sparsely localised in the xylem, without connections to the conduits (Figure 2). Interesting types of apotracheal parenchyma are initial and terminal parenchyma. Although the initial and terminal parenchyma are formed at the beginning and end of the growing season, respectively, both apotracheal parenchyma types are closely associated with growth rings and are localised alongside the border of an annual ring [43,44,45,46]. However, this xylem parenchyma classification is only used for angiosperms because, in coniferous wood, living cells are mostly represented by ray (radial) parenchyma and sometimes by axial parenchyma, as well as epithelial cells in some species (Figure 2). The epithelial cells in gymnosperms surround resin canals and produce resin, which helps coniferous plants to defend themselves against biotic agents after injury [18,47].

Presently, living cells are assumed to constitute a significant portion of the secondary xylem, in terms of both structure and function (Figure 2) [20,37]. Moreover, the xylem parenchyma in angiosperms is more abundant than that in gymnosperms and usually belongs to both the radial and axial systems. However, in angiosperm tree species, the total amount of parenchyma typically ranges between 20% and 40%, and the axial parenchyma fraction is highly variable, ranging from ≤1% to ≥30% (Figure 2). For example, representatives of the genera *Populus*, *Aesculus, Magnolia*, and *Eucalyptus* are characterised by sparse axial parenchyma, whereas the axial parenchyma is abundant in *Quercus*, *Robinia*, and *Ceiba* [46,48,49,50]. In contrast, conifers have fewer living xylem cells (between 7% and 10%), which may be limited to the ray parenchyma [1,18,29,47] (Figure 2). Moreover, global analyses of parenchyma fractions showed that the amount of living cells is determined by the climatic zone, with a greater number of xylem parenchyma cells found in tropical species than in temperate and subtropical woody species. Interestingly, these observations are linked to differences in the axial but not in the ray parenchyma fractions in angiosperm species [29]. Studies on one evergreen conifer species (*Juniperus thurifera* L.) showed that the abundance of ray parenchyma varies annually, suggesting that interannual changes in temperature and precipitation affect the volume of living cells in wood [51]. In addition, plants representing different growth forms show varied proportions of xylem parenchyma, being especially abundant in lianas and stems succulents [49,50,52,53,54]. For example, in the genus *Machaerium floribundum* Benth., which can exist as a tree growing up to 25 m in height and as a liana, approximately twice as much axial and radial parenchyma is present in the xylem of the liana form than in the tree specimens [52]. Remarkably, the amount of parenchyma tissue fraction in stem succulents can be as high as 70% [29,53].

**Figure 3:**
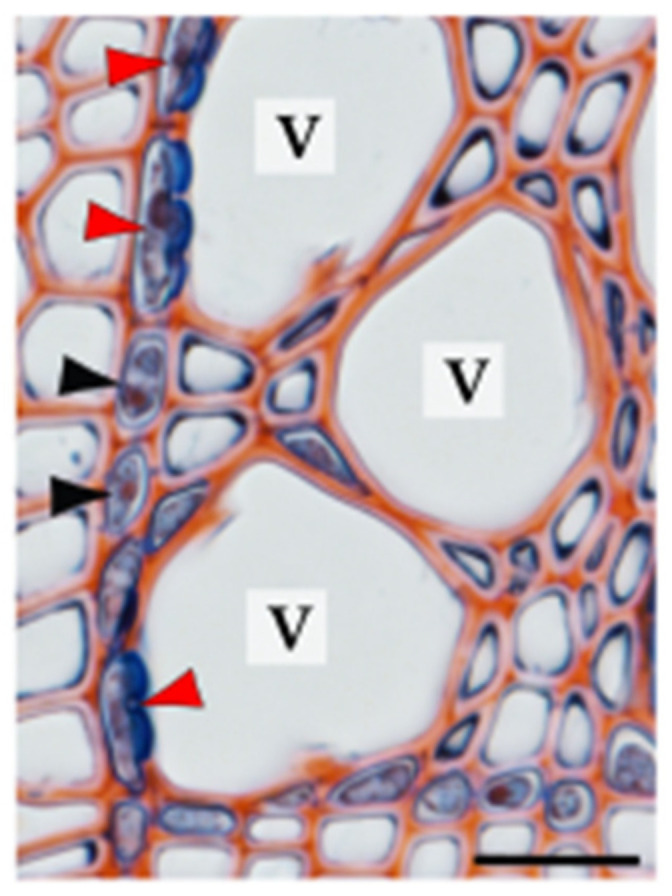
Vessel-associated cells (VACs) and isolation cells on a transverse wood section of *Populus* sp. VACs with contact pits are marked with red arrowheads, and isolation cells with black arrowheads. Abbreviation: V—vessel. Scale bar—20 µm.

The other xylem parenchyma classification system, which is based on living cell localisation with respect to conductive elements, identifies two types of xylem parenchyma cells: isolation cells and contact cells, both representing axial and ray fractions of the parenchyma (Table 1). Isolation cells have no direct contact with conduits and specialise in symplasmic transport because of the numerous plasmodesmata located in the simple pits, which connect them with other living wood cells [1,55,56,57]. In contrast, contact cells are linked with adjacent conductive elements via large contact pits and with neighbouring parenchyma cells, including isolation cells, via plasmodesmata gathered in the simple pits [1,23,56,58]. The biological term “contact cells” can be used for all xylem parenchyma cells that are in direct contact with the dead tracheary element, whereas vessel-associated cells (VACs) refer to the parenchyma cells connected to vessel elements and are thus used exclusively for angiosperms [58,59] (Figure 3). 

The region where contact pit links a contact cell/VAC with a tracheary element is devoid of plasmodesmata and lined with an amorphous layer. Therefore, the transport between living parenchyma cells and dead conduits cannot occur via the symplasmic pathway [24,60,61]. However, recent studies have experimentally demonstrated that solute uptake from dead conduits to VACs involves the endocytic pathway, including both clathrin-mediated and clathrin-independent processes [62]. At the ultrastructural level, contact cells are characterised by dense cytoplasm, numerous vesicles, well-developed endoplasmic reticulum, and abundant mitochondria and vacuoles. These cells, similar to other xylem parenchyma cells, can store starch in amyloplasts but in much smaller quantities [24,26,62,63]. Owing to the location and unique character of contact cells, their role in many processes occurring in wood has been recently studied, demonstrating their highly specialised nature (e.g., [28,62,64,65,66,67,68,69]).

Thus, xylem parenchyma cells are an important component of the wood bulk, crucial for the proper functioning of the tissue and the plant as a whole. In this review, we focus on the following selected roles of the xylem parenchyma in tree functioning, with emphasis on contact cells/VACs: (1) as a storage tissue; (2) in the regulation of xylem conductivity; (3) in a defence mechanism; (4) in the mechanical support of the wood. Differences between angiosperm and gymnosperm wood have also been accounted for, wherever possible. 

## 2. Xylem Parenchyma as a Storage Tissue

One of the primary roles of xylem parenchyma cells is their function as storage tissue (e.g., [69,70]). The pool of stored compounds depends on stem anatomical features, and a key variable in their storage ability is the abundance of the xylem parenchyma, usually the only cells that remain metabolically active in mature secondary xylem [26,43]. Since living xylem parenchyma cells are an essential element of wood, molecules such as water, non-structural carbohydrates (NSCs), and lipids stored in these cells constitute a large portion of accumulated compounds. Thus, the pool of stored compounds reflects the present condition of a plant, its phenology, the period of the year, and the impact of environmental factors on the plant (e.g., [71,72,73]). Overall, the compounds stored within the living xylem parenchyma play vital roles in the functioning of the whole plant and are particularly important for tree survival during stress periods. 

Generally, storage cells are xylem parenchyma cells that are distant from conduits, namely, isolation cells. Therefore, the characteristic feature of contact cells, that is, the absence of a large vacuole, indicates their limited storage capacity [24,58]. Although some compounds can be present in the xylem parenchyma cells that are in direct contact with conduits, they are still considered temporary storage sites [22,50,64,74]. Thus, the vast majority of examples presented in this section refer to isolation cells.

### 2.1. Water Storage

Xylem parenchyma cells can store water, which is essential for the proper functioning of a plant. Water stored in the living cells of wood is a fraction of intracellular water storage, which also includes the cells of the pith and the layers outside the vascular cambium, such as phloem [70]. Water stored in the xylem parenchyma cells is critical for the survival of both angiosperms and gymnosperms during stress conditions, such as drought (e.g., [75,76,77,78,79]). Under water stress, plants may withdraw water from storage tissues to sustain the functioning of the vascular system [70]. 

Plants that occur in water-poor habitats and rely on water stored in their stems present specific anatomical modifications. In such species, extra capacity for intracellular water storage can be provided by the increased number of unlignified, thin-walled parenchyma cells, which can be structurally diverse [70]. For example, *Adansonia* and *Bursera* form extra apotracheal bands of living cells because of the excessive proliferation of the axial parenchyma [55,80,81]. In addition, the enlargement of the liana stem is a result of the increased fraction of the xylem parenchyma. Thus, abundant living cells in the secondary xylem support water and nutrient storage [52,82,83]. Studies on tropical dry forest species have reported that the number and distribution of xylem parenchyma cells strongly influence plant performance under water stress; the species with extensive xylem parenchyma cell fractions were considered to be drought avoiders [84,85,86]. It has also been hypothesised that the remarkable amount of water stored in the parenchyma cells in the stems of the liana *Machaerium floribundum* Benth. may help in surviving through dry periods [52]. Moreover, *Malus* × *domestica* Gala (4×), which is characterised by an increased amount of living cells in wood, overall performed better during short and moderate drought stress conditions when compared with the ploidy variant Gala (2×), indicating the role of xylem parenchyma cells in water storage [87].

### 2.2. Carbon Storage

Carbon is known to be particularly important during carbon-demanding periods, such as leaf-bud breaking and shoot growth at the beginning of the growing season. Living wood cells are considered carbon storage sites. The most common carbon compounds that are stored in the wood tissue are NSCs, which are stored in both ray and axial xylem parenchyma [88,89,90,91,92]. Among NSCs, the most abundant are starch and low-molecular-weight sugars, such as glucose, fructose, and sucrose, and the less abundant, but still relevant in some tree groups, are oligosaccharides and polyols [69]. Therefore, the abundance of living cells strongly affects the NSC storage capacity of wood. A strong positive correlation between parenchyma cell content and NSC storage has been revealed in temperate angiosperms like *Aesculus hippocastanum* L., *Acer pseudoplatanus* L., *Fraxinus excelsior* L., *Fagus sylvatica* L., and *Quercus robur* L. [50,93]. Notably, coniferous wood, which is characterised by a smaller fraction of living cells than that in angiosperms, presents lower NSC concentration [94]. However, the correlation between the amount of the xylem parenchyma and carbohydrate accumulation in this tissue is complex, which is stronger for temperate species but not sufficiently distinct for tropical trees [50] and thus, requires further investigation. Although the carbohydrate storage capacity is dependent on the abundance of the parenchyma fraction in a given plant, carbohydrate production and employment cause for example seasonal fluctuations in NSC levels, which hinders the experimental assessment of the link between anatomy, that is, the amount of xylem parenchyma and of carbohydrates [26,89,95,96,97,98]. 

However, the storage capacity of the xylem parenchyma fraction is not always fully utilized [26]. According to the historical classification, plants that rely on NSCs as the main form of carbon storage (starch trees) can be distinguished from so-called fat trees, which accumulate natural lipids in the wood [99]. Some woody representatives of angiosperms and gymnosperms (e.g., *Pinus* sp., *Tilia* sp.) show higher amounts of stored lipids than those of NSCs [89,100]. Except for the role of stored carbohydrates during unconstrained plant growth, the increased amount of NSCs promotes drought resistance in plants. Various transgenic crop plants, which are engineered to accumulate an increased amount of fructan, possess enhanced drought tolerance, including better growth rate under stress conditions [101]. Furthermore, the manipulation of NSC concentration in the seedlings of 10 tropical tree species revealed that NSC enrichment increases stem water potentials and the overall tolerance for water deficit [102]. The involvement of stored NSC in promoting plant drought resistance is consistent with the fact that embolism repair is a carbon-demanding process. Therefore, it can be hypothesised that the increased amount of NSCs improves the process of embolism repair because NSCs are the main forms of carbon storage [102,103].

## 3. Regulation of Xylem Hydraulic Conductivity by the Xylem Parenchyma

**Figure 4:**
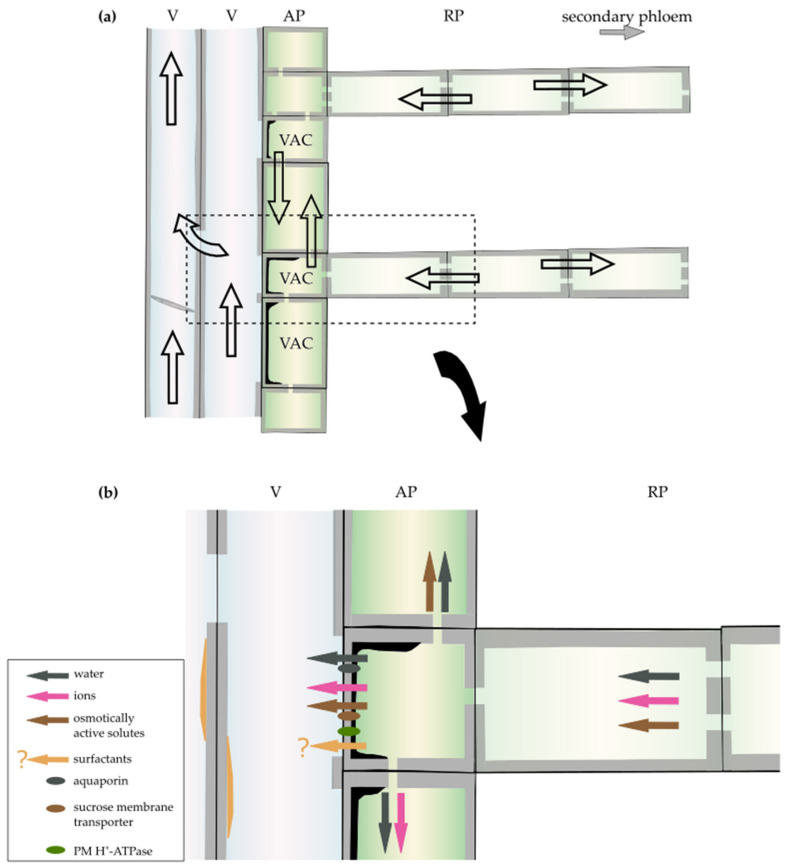
Schematic illustration of different transporting pathways and processes regulating xylem hydraulic conductivity operating in wood. (**a**) Major directions of cell-to-cell transport (open arrows) in angiosperm wood. The location of the secondary phloem is marked with a grey arrow. The area marked with a dotted line on (**a**) is magnified in (**b**). (**b**) Varied processes facilitated by parenchyma cells that are involved in the regulation of xylem hydraulic conductivity. Abbreviations: V—vessel, VAC—vessel-associated cell, AP—axial parenchyma, RP—ray parenchyma. The secondary cell wall is marked with grey, the amorphous layer in VACs with black, and the surfactants on the surface of vessel walls with orange colour.

Previous studies showed that xylem parenchyma cells have the potential to directly and indirectly modulate solute flow within tracheary elements (e.g., [28,93,104]). Thus, in this section, we discuss how the activity of the xylem parenchyma affects the axial transport of solutes within the xylem (Figure 4a).

### 3.1. Embolism Repair

Xylem sap transported within the interconnected system of dead tracheary elements under negative pressure (tension) is prone to cavitation [105], which can be induced by air seeding and leads to bubble formation when air passes through the pores of the pit membranes [106,107,108]. Cavitation can also be induced by air bubbles adhered to the cracks in the walls of dead elements [109,110,111]. Further expansion of the gas bubble causes embolism formation, which can spread within a single tracheary element and to other elements of the vascular system relatively quickly and easily. Studies have also reported that embolism can be induced by freezing under laboratory conditions [5,17,112]. Cavitation, and consequent embolism formation, not only occurs in leaves, roots, and stems [112,113,114] but also occurs in the secondary xylem.

By disrupting the xylem sap continuum, embolism reduces the xylem transport capacity, which may result in both short-term effects, including stomatal closure and reduction in the rate of photosynthesis, and long-term effects, such as growth reduction and even plant death [115,116,117]. Although the process of embolism repair is not completely understood, plants may reinstitute xylem functionality on both daily and seasonal bases, and it is generally accepted that embolism refilling may occur under tension [21,93,118,119,120,121]. Herbaceous plants mostly rely on root pressure to recover from embolism formation, whereas trees adopt more complicated strategies and rely on the activities of the xylem parenchyma and phloem. 

Embolism repair in trees is a three-step process, including (1) sensing embolism formation signals by contact cells/VACs, (2) generating an osmotic gradient between embolised conduits and adjacent living cells, and consequently creating a driving force for (3) water movement into the tracheary elements. 

Although few hypotheses on the first step of embolism repair, sensing, have been proposed, this phenomenon remains unclear. Previous studies on laurel (*Laurus nobilis* L.), wherein sonication was used to mimic cavitation, suggest that embolism repair is triggered by vibrating tracheary element walls during cavitation, which is sensed by VACs [122]. Moreover, sonication induced starch depolymerisation in the VACs, which is an indispensable part of the xylem’s recovery from embolism. This suggests the biophysical nature of the signal initiating embolism repair, which was later called mechanosensing [122]. Another hypothesis suggested that sucrose or other low-molecular-weight carbohydrates are the signals triggering embolism repair, which normally leak from the xylem parenchyma and phloem to the xylem sap and start accumulating in the conduit cell walls when embolism occurs [123]. Other studies showed significant changes in the expression of genes involved in carbohydrate metabolism upon embolism sensing by parenchyma cells surrounding the xylem vessels. Furthermore, the presence of air in the embolised vessels supposedly triggers the initial response via a reduction in hypoxic stress and ROS formation [124].

In the second step of embolism repair, an osmotic gradient between the lumen of the embolised conduit and the adjacent parenchyma cell is generated [125], likely by the transport of solutes from these neighbouring xylem parenchyma cells. These solutes are suspected to be too large to pass through the interconduit pits and are held in the tracheary elements to which they were transported. Thus, a generated high solute concentration retracts water from the living wood cells and the transpiration stream to deliver it to the cavitated tracheary elements [118]. Substances that are indicated as the source of the gradient are carbohydrates stored in the xylem parenchyma cells (e.g., [93,126]) (Figure 4b, brown arrow). During winter, when no starch is present in the VACs of laurel, no xylem recovery from experimentally induced embolism was detected, suggesting that starch-to-sugar conversion is necessary to generate a driving force for water transport into embolised conduits [127]. Additionally, starch levels in xylem parenchyma cells decrease with progressing conduit refiling in *Populus trichocarpa* and *Laurus nobilis* [27,123]. Moreover, the inhibition of xylem refiling not only stops the depolymerisation of starch in VACs [66,68] but also up-regulates genes, including those coding for enzymes related to carbohydrate metabolism and starch degradation, as was demonstrated in *P. trichocarpa* [124]. 

Based on significant changes in the expression of genes involved in carbohydrate metabolism after embolism formation in *P. trichocarpa*, it was hypothesised that modifications in xylem parenchyma cells promote the release of sucrose from starch, which is needed for the refiling process [124]. Previous studies showed that in *Juglans regia* L. (walnut) trees, the sucrose membrane transporter might be directly involved in embolism recovery, particularly during freeze-induced embolism, as the JrSUT1 level was upregulated right after the freeze–thaw cycles over the autumn–winter period [25]. Simultaneously, JrSUT1 and plasma membrane H^+^-ATPase have both been localized in xylem parenchyma cells adjacent to vessels, indicating the presence of an H^+^/solute co-transport mechanism [25] (Figure 4b, brown arrow and green oval). All these studies indicate the role of carbohydrates stored in xylem parenchyma cells in embolism repair, which are transported to the adjacent tracheary elements to generate an osmotic gradient for xylem refilling during embolism (Figure 4b, brown arrow).

Furthermore, the process of conduit refilling in the xylem has been associated with phloem unloading. Over the past decades, phloem has been considered an additional source of sugars and other solutes, which drive the osmotic activity for water movement, and ray parenchyma is considered a potential pathway for their transport into the embolised conduits [68,111,119,126,127] (Figure 4b, brown arrow). The importance of the radial transport via xylem rays was demonstrated using girdling experiments, which indicated that when the phloem region was removed, the capacity to restore losses in hydraulic conductance was also reduced [119,127,128]. Additionally, it was suggested that the depolymerisation of starch in the xylem parenchyma is triggered by signals transferred from the phloem [127], or that the decreasing starch levels in the xylem parenchyma cells make them a strong sink and trigger phloem unloading and active transport of sugars to the xylem [27] (see more details about the role of phloem in embolism recovery in [68]).

The third step in the embolism repair process is water movement. When an adequate driving gradient is generated, osmosis occurs to refill the embolised tracheary elements with water released into the lumen of conduits from the adjacent xylem parenchyma cells [125] (Figure 4b, dark grey arrow). The high levels of plasma membrane aquaporins (membrane water channels) in VACs probably improve water transport capacity, as changes in the membrane water permeability during the refilling process are related to an increased expression of aquaporins in VACs in *Juglans regia* and *Populus trichocarpa* [66,129] (for a review on aquaporins, see [28], Figure 4b, dark grey oval). Empirical studies using fluorescent dyes suggest that the radial water transfer from outer layers (phloem) to the embolised xylem in *Eucalyptus saligna* is facilitated by the symplast of ray parenchyma cells [40]. Moreover, studies suggest that water stored in the bark of *Castanea dentata* (American chestnut) is redistributed to the xylem via ray parenchyma early during drought stress to minimize the risk of hydraulic failure [130] (Figure 4b, dark grey arrow).

In addition to the three-step embolism repair hypothesis, other mechanisms for embolism repair have been suggested. One proposed mechanism hypothesises that xylem refiling is based on the pressure generated by the expansion of the living tissue surrounding the xylem vessels. According to this hypothesis, parenchyma cells first lower their osmotic potential using starch hydrolysis, which, in turn, increases their turgor pressure by water influx and consequently creates pressure for conduit refilling with water transported from the cells adjacent to the tracheary elements [131,132]. However, this hypothesis has been questioned many times and is no longer studied [111,133]. 

Another interesting postulate on xylem refiling, emphasizing the role of root pressure, was proposed by Westhoff et al. [134,135]. The group tested vessel refilling by measuring seasonal changes in osmolality in the xylem conduits of *Betula pendula* Roth (birch) trees, with special attention given to spring refilling. They reported that root pressure is an important driving force for the refilling of birch vessels, and the development of root pressure coincided with the appearance of osmolality gradients, suggesting that in tall trees, xylem conduit refilling relies on a dual mechanism that operates from the base (by root pressure) as well as from the top (by hydrostatic pressure generated by xylem-bound osmotic pressure) of the tree. In the proposed dual mechanism, refilling of vessels is initiated by root pressure and followed by the generation of a hydrostatic pressure gradient that results in the radial movement of water into the empty vessels. Additionally, it is likely that sugars are directly released into the xylem and that starch is enzymatically degraded, indicating the role of living xylem cells as a source of water and sugars [134,135].

The sequence of events during hydraulic recovery from xylem embolism has not been fully elucidated and can be partially explained by the heterogeneity of mechanisms that dominate in different species. Therefore, the involvement of xylem parenchyma cells in xylem refilling suggests that the process of embolism recovery must correlate with parenchyma abundance, which was later confirmed using anatomical analyses, vulnerability to xylem embolism, and measurements of dehydration time on excised branches from 12 angiosperm species [93]. The results strongly suggested that embolism reversal was more effective in species with a higher proportion of parenchyma cells and that their abundance may be an important source of carbohydrates and/or water [28,93]. Additionally, the assumption that xylem refilling relies on the living cells of the wood and phloem verified the hypothesis that conifers have a lower capacity for embolism refilling, as these species are characterised by lower parenchyma fractions than those in angiosperms [28,94,136]. Thus, the mechanism of conduit refilling in gymnosperms is still ambiguous and generally less studied. Although spring refilling of winter embolism has been documented in *Picea* and *Abies* species (e.g., [137,138,139]), data on refilling during a short timescale are limited. Moreover, it has been postulated that refilling might not occur on a daily basis in conifers [136]. Comparative analyses of angiosperm and gymnosperm species to determine the correlation between the key structural and functional traits associated with embolism vulnerability verified that different mechanisms enable xylem refilling in the two plant groups [140]. Thus, unlike gymnosperms, angiosperms have an embolism-reversal capacity, wherein parenchyma plays an important role, and such autonomy from xylem embolism repair mechanisms in conifers may result from the presence of interconduit torus–margo pits, which prevent the spread of air from embolised tracheids [18].

### 3.2. Ion-Mediated Increase in Xylem Hydraulic Conductance

The ion-mediated increase in the xylem hydraulic conductance explains how plants spatially regulate the internal solute flow. Although this process is poorly understood, the living xylem parenchyma, which is a source of various compounds, affects the properties of xylem sap and, consequently, xylem hydraulic resistance (e.g., [141,142,143,144,145]). The ion-mediated increase in hydraulic conductance of xylem was first described by Zimmermann in 1978, who observed a significantly increased hydraulic conductance in *Acer saccharum* Marsh. (sugar maple) stem segments immediately after distilled water was replaced with tap water. Zimmermann attributed this phenomenon to the swelling and shrinking of the vessel-to-vessel pit membranes owing to the variations in osmotic strength of the flowing solution, without any experimental evidence [146]. Later, studies on the herbaceous perennial *Dendranthema* × *grandiflorum* Tzvelev (chrysanthemum) identified that the increased xylem hydraulic conductance was because of the changes in the fluid composition and the presence of cations in the flowing solution [143]. 

Ion-mediated flow enhancement was further verified in 19 angiosperm and 5 conifer species, with the enhancement ranging from 1.1 times in conifers to 2.5 times in some angiosperm species. Moreover, it was suggested that the process was mediated by changes in the intervessel bordered pits [144], where pit membranes provided significant hydraulic resistance to the flow of xylem sap, which was shown to respond differently to sap ion concentrations [147]. Pectins, also known as hydrogels, are the main components of intervessel bordered pits and are poly-electrolytic in nature. Owing to ionic interactions, hydrogels are capable of swelling and deswelling, affecting the hydraulic resistance of xylem [144]. In tobacco plants, the thickness of the pit membranes was modified by varying the ionic composition of the surrounding solution [147]. Therefore, it can be assumed that the properties of the solution within conduits affect xylem conductivity via the modification of pit membranes. 

The involvement of an ion-mediated increase of xylem hydraulic conductivity is a universal mechanism that compensates for the cavitation-induced loss of xylem conductivity. In laurel, an increase in K^+^ concentration and the osmolarity of xylem sap was triggered by xylem cavitation [111,141], which was subsequently confirmed in *Prunus laurocerasus* L., *Phytolacca dioica* L., *Ceratonia siliqua* L., *Persea gratissima* L., and *Olea europaea* L. [142,148]. In three Mediterranean evergreen species, the ionic effect was utilized by plants to alleviate the impact of xylem embolism, indicating that the integration of the refilling of xylem conduits and of the ion-mediated enhancement of stem hydraulic conductance is important for a plant’s response to drought [148]. Moreover, contact cells/VACs, with direct access to conduit lumina, have been proposed to be the source of ions; previous studies on ion-mediated increases of xylem hydraulic conductance suggest that this phenomenon requires metabolically active cells [104,149] (Figure 4b, pink arrow). Immunolocalization in *Robinia pseudoacacia* showed that plasma membrane H^+^-ATPase was much more abundant in VACs than in other xylem parenchyma cells [150]. The increased activity of H^+^-ATPase in VACs can create high activity of metal ions, which, in addition to a lower apoplastic pH, results in the release of inorganic ions into the xylem sap [24] (Figure 4b, green oval and pink arrow). Moreover, the molecules released into the conduits can be delivered from the phloem to the contact cells/VACs via ray parenchyma (e.g., [38,68,148,151]). The radial movement of minerals via rays in trees has been experimentally demonstrated in *Cryptomeria japonica* D. Don (Japanese cedar) using an isotope of caesium (Cs) and the freeze–thaw method to kill living wood cells [152]. Additionally, the importance of the radial movement of molecules via rays for xylem conductivity has been shown in maple species, in which a decrease in the hydraulic conductance of the xylem resulted from the reduced ion transport from phloem girdling [151] (Figure 4b, pink arrow).

### 3.3. Surfactants

Recently, new agents called surfactants, which reduce the surface tension of water, have been identified to influence the internal flow within the xylem [153,154,155]. The presence of an artificial surfactant in the xylem sap strongly alters xylem vulnerability to cavitation in both conifer and angiosperm species [156,157]. Furthermore, surfactants have been found in the xylem sap of woody representatives of major angiosperm clades [153,154,155,158]. In situ studies by Losso et al. [159] on hydraulic safety in conifers (*Picea abies* and *Pinus mugo*) indicate that solutions with low surface tension cause higher vulnerability to drought-induced xylem embolism [159]. In contrast, Schenk et al. [153,154,155] hypothesised that insoluble lipid-based surfactants increase the hydraulic safety in angiosperms, mostly by coating hydrophobic surfaces and nanobubbles, and thereby keeping the latter below the critical size at which bubbles expand to form embolism [153,154,155]. A similar mechanism of surfactant action was observed in *Populus nigra* L., and it was suggested that woody tissue assimilates play a role in the synthesis of xylem surfactants, which is facilitated by VACs [160]. The differences in surfactant effects may result from varied mechanisms regulating xylem hydraulics in gymnosperms and angiosperms. However, surfactants found in the xylem sap probably originate from contact cells/VACs [24,153,154,155] (Figure 4b, orange arrow).

## 4. Role of the Xylem Parenchyma in Defence Mechanism

Long-living organisms, such as trees, are exposed to numerous biotic and abiotic stresses, the effects of which accumulate throughout their lifespan. Hence, an efficient defence mechanism based on compartmentalisation, limiting, or curtailing the spread of an infection or injury, is important for the survival of woody species [161]. Thus, several efforts have been made to understand plant defence responses against various stressors. In this section, we emphasise the role of living wood cells in the defence mechanism of trees. 

### 4.1. Compartmentalisation of Decay in Trees (CODIT) Model

The CODIT model was developed in the 1970s, based on numerous observations of the plant defence responses against infections caused by decaying fungal pathogens [161,162]. The model assumes that defence mechanisms in big woody organisms rely on anatomical compartments that exist in the secondary xylem or those that are created immediately after wounding to prevent the spread of a pathogen [161,162,163]. Subsequently, an expanded version of the CODIT model was proposed, according to which, compartmentalisation was used in a much broader sense, including the responses to other biotic (e.g., herbivores, insects) and abiotic (e.g., damages caused via wind and snow) factors [163,164,165,166]. The latest interpretation of the CODIT model applies to responses to any desiccation-inducing phenomenon [167]. Therefore, the postulate that “D” in the CODIT model should stand for “Damage” or “Dysfunction” rather than only for “Decay”, as in the original model, seems fully entitled [163,164,165,166]. 

According to the CODIT model, a plant’s reaction to a wounding factor can be viewed as a two-part process, which is based on the so-called four “walls” [161]. The xylem parenchyma cells play a fundamental role in limiting the spread of the damage, as they are involved in each of the four walls of the CODIT model [161,163]. The walls are a representation of the anatomical compartments, which (1) are already present at the time of wounding in the secondary xylem and form a reaction zone (Part I, Walls 1–3), and (2) are formed after wounding, creating a barrier zone (Part II, Wall 4). Notably, the walls in the CODIT model have a gradable barrier strength, with Wall 1 being the weakest and Wall 4 being the strongest [161,163]. 

In Part I, Wall 1 relies on VACs, which are involved in vertical blockage formation within the vascular elements, Wall 2 relies on axial parenchyma cells that prevent inward and outward spread of the damage, and Wall 3 relies on the ray parenchyma that blocks lateral spread. Part I is also referred to as passive resistance because it relies on naturally occurring compartments of the wood [161,167,168]. In contrast, Part II, or active resistance, is activated after damage, triggering the formation of Wall 4, which creates a specific barrier zone formed by the rings of xylem parenchyma cells that are produced by the cambium meristem after wounding. Thus, Wall 4 is responsible for the separation of the infected wood region from the healthy zone and the newly produced secondary xylem [168,169].

As xylem parenchyma cells play a fundamental role in the defence mechanism in trees, the CODIT model will be described here in more detail. The first barrier in the model relies on the presence and activity of VACs (Wall 1 of the CODIT model) and includes conduit occlusion via the deposition of gums and/or the formation of tyloses [170]. Gums are amorphous materials that are water-insoluble, which occlude conduits when produced and secreted by VACs and represent a wide range of substances, such as polysaccharides, including pectins [170,171,172,173]. Tyloses are defined as thin- or thick-walled outgrowths of VACs that expand into the tracheary lumen via contact pits and may contain inclusions in the form of storage products (e.g., gums, starch, resins). They may form secondary walls around them when their expansion is completed [44,170,171,174]. Additionally, the cell walls of tyloses are encrusted with antimicrobial compounds [175] increasing the effectiveness of preventing the spread of the pathogen, and some tyloses are suberized, as in, for example, *Populus basalmifera* L., *Ulmus americana* L., and *Quercus rubra* L. [176]. In *Vitis vinifera* cv. Chardonnay, the vascular blockage reaction in VACs via the development of tyloses is rapid after wounding [177]. Tyloses are typically formed in the vessels of angiosperm, but they may also develop in the tracheids of conifers from ray parenchyma cells, as observed in *Pinus elliotti* Engelm. and *Pinus taeda* L. [178,179]. However, the first two walls (Walls 1 and 2) in some conifer species are rather based on tylosoids, the products of the epithelial cells that surround and clog resin canals and prevent the spread of the decay within the reaction zone. Hence, it is used to describe resin canal blockage by epithelial cells in conifers as a substitute for tyloses in angiosperms [1,167].

Furthermore, the type of vascular occlusion depends on the season; gums are formed in winter, whereas tyloses are formed in summer and autumn [173]. However, seasonal variation in the type of vascular occlusion is a rare phenomenon, and the quick response resulting in the formation of conduit occlusions via tyloses and gums was initially considered a response to indirect embolism-causing factors [180,181]. Nevertheless, the present CODIT model is applicable to all responses to all desiccation-inducing phenomena, as conduit occlusion is a part of the stress response when it appears after wounding. 

To limit damage propagation, trees use diverse strategies within Walls 2 and 3, which are based on the axial and ray parenchyma. One of them involves the production of various substances that act as phytoalexins (e.g., phenolic compounds, elemental sulphur, biphenyl, dibenzofuran) in the living xylem in response to the wounding factor [161,182,183,184,185]. Additionally, various antimicrobial compounds, such as flavonoids, and suberin can be synthesised from NSCs stored in xylem parenchyma cells, mostly isolation cells [167,186,187,188]. Suberin deposition is important against microbial infections, as this compound is toxic to microbes. Thus, the formation of a water-repellent fatty layer, owing to suberin deposition, plays an important role in the effectiveness of compartmentalisation after wounding [168,189]. However, suberization in response to infection or injury may be weaker in conifers than in hardwood species because of the fewer parenchyma cells in conifer wood [190]. Moreover, trees are more susceptible to fungal pathogens in the dormant season (winter), as the above-mentioned compounds are not synthesised [191,192,193]. 

Secondary metabolites (SMs) are also synthesised in the xylem parenchyma in response to damage. These metabolites accumulate in the xylem parenchyma cells and are released to infiltrate intercellular spaces within the reaction zone to retard the spread of the pathogen [167,187,194,195]. In addition, the living cells in the reaction zone previously reinforced with suberin frequently undergo programmed cell death (PCD) and form a layer of dead parenchyma cells filled with antimicrobial compounds [167]. 

According to the CODIT model, the last wall (Wall 4), also called the barrier zone, separates and secures newly formed secondary xylem and is composed of rings of parenchyma cells, formed after infection or injury. This wall was first described in elm (*Ulmus* sp.) trees as a layer of flat parenchyma cells filled with starch [169,196]. In *Quercus robur* L., up to 30-cell layers of axial parenchyma have been described in the barrier zone, where the cells frequently undergo suberization [197,198]. The increased amount of suberin is responsible for the strength of Wall 4 [194,198,199]. Moreover, in *Eucalyptus* species, special tissue called wound wood may be produced in the vicinity of damaged secondary xylem, characterised by increased parenchyma density and a specific composition of SMs. Therefore, a wound wood is assumed to be formed prior to the production of the secondary xylem and is considered to be an effective mechanism to restrict the outward spread of fungal decay [200]. 

### 4.2. Heartwood Formation 

Conduit blockage, owing to the formation of tyloses and secretion of gums by contact cells/VACs, occurs also naturally, not only in response to a wounding factor but also as a part of the sapwood to heartwood transition that accompanies plant ageing [170]. Moreover, during conduit blockage, for example, in *Cryptomeria japonica* and *Juglans nigra* L., various antimicrobial SMs are released from parenchyma cells prior to PCD and, consequently, accumulate in the heartwood [201,202]. These chemical changes make heartwood more microbe-resistant than sapwood, in which toxic compounds impregnate the cells and limit the spread of the infection [203,204]. Thus, the pathogen-resistant characteristics of heartwood result from the activity of the living xylem parenchyma cells. 

## 5. Parenchyma Cells Contribute to the Mechanical Properties of Wood

### 5.1. Turgor Pressure of Xylem Parenchyma Cells

Xylem parenchyma cells can store large amounts of water (e.g., [43,70,84,86]). Therefore, the stiffness of parenchymatous tissue is affected by changes in cell water content, indicating that xylem parenchyma cells probably play a mechanical role [43,205,206]. The structural role of parenchyma cells has been studied in six species of baobab trees from Madagascar (*Adansonia* ssp.) [53], and it was reported that baobab wood contains a significant proportion of parenchyma cells in the main stem (69–88% of its volume), which increases the stem water content (0.57–0.79 in water volumetric fraction). Additionally, the wood of baobab trees is characterised by a low lignin content (6–9% of dry mass) and the presence of only a few thin-walled fibres, which are scattered within the wood region, suggesting that the wide stems of these plants predominantly serve as water storage sites (e.g., [207,208]). However, as the use of stored water is limited in baobab trees, whether these plants strongly rely on water accumulated in their stems is debatable [209,210]. Additionally, anatomical studies and bending tests in several species have shown that xylem parenchyma plays a key role in mechanical support [53], and morphology similar to baobab trees in stem succulents (e.g., *Idria collumnaris*, *Pachycormus discolor*, *Bursera microphyIla*) (without experimental evidence) indicates that the abundance of parenchyma cells and high stem water content in their stems is also crucial for biomechanical properties [53,81,211]. 

### 5.2. The Role of Xylem Rays for the Mechanical Properties of Wood

Xylem parenchyma cells that run radially in trees form xylem rays. Depending on the tree species, their proportion typically ranges between 8% and 40% of the entire wood, in extreme cases, even only 4% in some coniferous species, such as *Pinus*, or up to 80% in selected angiosperm species, such as *Adansonia*. Conifers have narrow rays, 1 or 2 cells wide (if they do not contain resin canals), whereas angiosperm rays are much wider, commonly from 2 to 6 cells wide but may reach up to 15 or more cells in *Quercus* sp. [48,212]. The radial strength of wood is markedly higher than its tangential strength in trees. Therefore, it is hypothesised that xylem rays play a key role in the maintenance and regulation of the radial strength of wood [213,214]. Although fewer studies have been conducted on a small number of tree species, the mechanical contribution of xylem rays has been experimentally verified [212,215,216]. 

The radially oriented ray parenchyma secures the inner architecture in both angiosperm and conifer species by acting as stiff pins and by preventing the layers of different stiffness from slipping off of each other. This function of rays is observed during plant bending (for example, as a consequence of strong wind), when the rays interlock the adjacent tree rings, limiting the shear stress [217]. Scanning electron microscopy of fracture initiation and propagation in *Picea abies* (L.) Karst. (Norway spruce) verified these observations, and it was hypothesised that rays impede the separation of cells along the growth ring borders caused by transverse tensile strains [218]. Furthermore, the significance of the radial strength of rays, particularly their orientation and volume, was shown in *Fagus sylvatica* L. [219].

Under artificial radial tension, rays change their orientation and run parallel to the radial force, which is consistent with the naturally observed increase in relative ray volume in tree parts stressed radially, indicating that ray parenchyma is mechanically adapted tissue [219]. These observations were subsequently verified via direct measurements of strength using microtensile testing, wherein isolated dry xylem rays were reported to be approximately 3 times stronger than the entire wood in its dry state [212]. Moreover, the importance of rays in the maintenance of wood structure was indicated when the mechanical properties of the wood of *Quercus robur* L. (oak) and *Fraxinus excelsior* L. (ash) were evaluated, as an individual ash tree with a higher fraction of ray volume was characterised by higher radial tensile strength [216]. Additionally, Ozden and Ennos [215] demonstrated that ash wood, characterised by a greater percentage and homogenous distribution of ray cells compared with those in silver birch (*Betula pendula*) and wild cherry (*Prunus avium*), is tougher and more resistant to fracture [215]. It has also been hypothesised that abundant xylem parenchyma cells in lianas may confer greater flexibility and, hence, resistance to twisting and girdling. However, these results are not strictly related to ray parenchyma but to the whole xylem parenchyma fraction [52]. Therefore, living parenchyma cells, especially those that are radially oriented, are important for wood functioning owing to their mechanical properties.

## 6. Final Notes

In this review, we discussed the scientific evidence that indicates the role of xylem parenchyma cells in tree functioning, particularly the experimental evidence suggesting that xylem parenchyma (1) strongly influences the storage ability of the secondary xylem; (2) is crucial for embolism repair, as sensory cells, and by generating the force and facilitating water movement; (3) serves as the source of ions during the process of ion-mediated increase in xylem hydraulic conductance; (4) serves as a potential source of surfactants for the reduction of water surface tension to influence the process of cavitation; (5) prevents the spread of wounding factors by functioning as a barrier in all the walls defined in the CODIT model; (6) has mechanical supporting properties. 

Although the understanding of the role of xylem parenchyma cells in the functioning of wood and whole trees is improving, many aspects require further investigation. Secondary xylem is still considered difficult to study, especially because of its location deep inside the plant and the presence of thick, lignified cell walls (e.g., [220]). In studies related to parenchyma, aspects associated with tree phenology are also hindering. One of the particularly common difficulties is the seasonality of woody plants. Seasonal variation is reflected in the ultrastructure and activity of xylem parenchyma cells [221,222,223] and thus, in the processes in which these cells are involved [152,222,224,225]. For example, seasonal changes in the pH of xylem sap (e.g., [150,226,227,228,229,230,231]) or in ion-mediated increases in xylem hydraulic conductivity [224] are well-documented in various species, and xylem parenchyma cells, namely, contact cells/VACs, play a crucial role in these processes. Therefore, seasonal variations and periodic phenomena, such as droughts, must be accounted for while designing experiments and data interpretation in studies related to the function of xylem parenchyma cells. 

A study on the relationship between the amount and spatial arrangement of xylem parenchyma and vessel diameter was conducted with more than 2000 woody angiosperm species, and it demonstrated that species with wide vessels have a high fraction of axial parenchyma tissue that is packed around them [49]. Similar comprehensive wood analyses of plant groups or species would help to determine the variability of the processes in which xylem parenchyma cells are involved.

In recent years, there has been a growing interest in living wood cells, and the analytical methods are becoming increasingly advanced. Present studies on woody tissues not only involve anatomical and developmental analyses (e.g., [232,233,234]) but also involve more sophisticated methods, including immunolocalization (e.g., [62,235,236]), tracer loading (e.g., [40,62,152]), and advanced imaging (e.g., [21,237]). Furthermore, owing to the progress in sequencing techniques and the introduction of next-generation sequencing (NGS) [238], genome-wide analyses of various tree species (*Populus* was the first tree species to be sequenced [239]) are becoming easier and more accessible. An example of the outcome of such a genome-wide study directly related to the wood-forming tissues in *Populus tremula* L. is the recently developed interactive tool called AspWood, which was constructed using RNA-seq data. This tool can be accessed freely to explore the expression profiles and co-expression networks in various cells of *Populus* wood [240].

There is also a database called PopIndels, which is a functional genomic tool created using the population of interspecific hybrid poplars carrying indels and the characterisation of these mutations using next-generation sequencing. It can be accessed to search for indels covering specific genes and to link phenotypes to regulatory genes [241]. Recently, a protocol for laser capture microdissection (LCM) of xylem tissues of woody plants has been developed [242], which, along with transcriptomic and metabolomic analyses, was employed on Norway spruce wood to determine the gene set expressed specifically in developing ray parenchyma cells [243]. This approach provides solid evidence of ray cells’ contribution to lignification of the developing tracheids, which is achieved by synthesising monolignols and producing the enzymes acting in the polymerisation step [243]. LCM was recently also used in a high spatial-resolution metabolomic study, where specific patterns of metabolites within the wood-forming zone of *Populus tremula* were determined [244]. As *Arabidopsis thaliana* has become an informative model for wood research (e.g., [245,246,247,248,249,250]), further development in innovative techniques for trees and the usage of Arabidopsis will facilitate better understanding of the regulation and functioning of wood, especially the xylem parenchyma, at the genetic level. This may streamline the economic utilization of wood, as the molecular tools will result in increased production efficiency of the raw material. 

## Figures and Tables

**Table 1:** Xylem parenchyma glossary.

**axial parenchyma**	parenchyma cells of a longitudinal system; usually form strands of axially elongated cells
**paratracheal parenchyma**	axial parenchyma neighbouring tracheary elements
**apotracheal parenchyma**	axial parenchyma not neighbouring tracheary elements
**ray parenchyma**	parenchyma cells of a radial system, grouped into radially oriented rays
**marginal parenchyma**	parenchyma bands at the ends of growth rings; associated with the end (**terminal parenchyma**) or with the beginning (**initial parenchyma**) of a ring
**contact cell**	axial or ray parenchyma cell in direct contact with tracheary elements via specialized pits (contact pits)
**vessel-associated cell (VAC)**	contact cell in direct contact with vessel elements
**isolation cell**	axial or ray parenchyma cell without direct contact with tracheary elements

## Data Availability

Not applicable.
